# A clustering-based approach to characterize autonomy profiles among multiple sclerosis patients: an application of the Qluster method in the FOCAL-MS2 study

**DOI:** 10.1186/s41687-026-01074-5

**Published:** 2026-05-25

**Authors:** David PAU, Alexandre Civet, Cécile Donzé, Géraud Paillot, Claude Mekies, Mikael Cohen, Lucie Brechenmacher, Catherine Mouzawak, Patrick Vermersch, Cyril Esnault, Svetlana Le Ralle

**Affiliations:** 1 Science, Roche SAS, Paris, France; 2 Hôpital Saint-Philibert, Lomme, France; 3 Association Aventure Hustive, Saint-Malo, France; 4 RAMSAY Clinique des Cèdres, Toulouse, France; 5 Université Côte d’Azur, Nice, France; 6 Structure régionale neuro SEP SYNAPSE, Le Vesinet, France; 7 University of Lille, Lille, France

## Abstract

**Background and objectives:**

Many Multiple Sclerosis (MS) patients have symptoms that impact on their autonomy, defined as being able to perform the roles that are most important to oneself, with or without help. This research aimed to identify distinct phenotypic autonomy profiles among People with MS (PwMS) and evaluate their 1-year longitudinal trajectories using the newly developed 10-dimension Multiple Sclerosis Autonomy Scale (MSAS).

**Methods:**

Data were drawn from the prospective, non-interventional FOCAL-MS2 study following 199 PwMS in France over 12 months. To uncover patient profiles, we applied an unsupervised clustering method using clinical and social parameters at baseline. Cluster stability was validated via bootstrap resampling and the Jaccard similarity index. Within-patient 1-year changes in MSAS subscales were evaluated against a Minimum Clinically Important Difference (MCID) threshold.

**Results:**

The analysis yielded six highly stable patient clusters (Jaccard indices ranging from 0.90 to 0.98). Cluster 1 (*n* = 30) comprised middle-aged patients on sick leave who were severely impacted regarding activities with others. Cluster 2 (*n* = 42) was characterized by a younger population (40–49 years) requiring occasional walking assistance. Cluster 3 (*n* = 42) consisted of middle-aged patients who maintained high levels of personal activities. Cluster 4 (*n* = 30) represented the oldest (≥60 years) and most disabled group at baseline; paradoxically, this subgroup showed an improvement in autonomy across most dimensions over the year. Finally, Cluster 5 (*n* = 19, without activities) and Cluster 6 (*n* = 36, professionally active women) both represented younger patients (<40 years) exhibiting high functional autonomy at baseline.

**Conclusion:**

Our study identified six unique clusters that differ not only in their baseline symptom burden but also in their 1-year trajectory of autonomy. This underlines the necessity to address specific socio-professional and psychosocial dimensions of autonomy unique to each patient’s profile.

**Supplementary information:**

The online version contains supplementary material available at 10.1186/s41687-026-01074-5.

## Introduction

Multiple Sclerosis (MS) is an inflammatory and degenerative demyelinating disease affecting the central nervous system. Its causes are partially unknown, and its evolution is difficult to predict. In France, approximately 135,000 people are affected by this condition [1]. The non-interventional FOCAL-MS2 study [2] aims to validate the psychometric properties of the Multiple Sclerosis Autonomy Scale (MSAS) comprising ten dimensions, focusing on patients’ daily activities and quality of life to better assess the disease’s impact on the patient’s autonomy.

Autonomy in MS is complex and generic tools do not consider certain dimensions of autonomy such as the importance of physical, emotional and functional autonomy. In a previous study [3], in order to address this need for autonomy assessment in the MS population, a new definition of autonomy was proposed. Based on 20 interviews and analysed according to the principles of the grounded theory (an inductive methodology that builds theory from data) and social role theory (which examines how social expectations define behavior), the autonomy was defined as “Being able to fulfill the roles that are perceived as most important to the patient (working, being a parent, helping others … ), with or without support”. To assess the level of autonomy of patients suffering from MS, the Multiple Sclerosis Autonomy Scale (MSAS) questionnaire was developed [3].

The objective of the FOCAL-MS2 study was to confirm the psychometric properties of the short item version of the MSAS questionnaire, including consistency, construct validity and reliability. Patients’ characteristics included in the FOCAL-MS2 study are available in [2].

The exploration of health data through clustering algorithms [4, 5, 6] allows for a better description of populations of interest by identifying the sub-profiles that compose them, thereby enhancing medical knowledge. Data clustering is a difficult task for many data scientists who are faced with a large literature and a large number of algorithms and implementations. To address this, the Qluster method, a generic, easy-to-implement, and robust workflow for clustering health data, had been proposed by Esnault et al. [7].

This article presents the application of the Qluster method to the patient population of the FOCAL-MS2 study. The primary objective of this research was to identify potential distinct groups of patients presenting similar characteristics to gain a deeper understanding of the disease’s impact on their autonomy and to reinforce medical knowledge. The secondary objective was to determine whether these distinct groups are associated with significant changes in the autonomy score over time.

While the exploration of health data through clustering algorithms allows for a better description of populations of interest, existing clustering studies in the MS field have predominantly focused on stratifying patients based on clinical disease courses, physical disability trajectories, or MRI features [8]. Although valuable for medical management, these traditional clinical phenotypes often fail to capture the subjective, psychosocial, and daily life impacts of the disease. Profiling People with MS (PwMS) based directly on their perceived autonomy—measured via specific Patient-Reported Outcomes (PROs) rather than standard clinical metrics—remains a significant gap in the literature. This analysis aimed to fill this unmet need by uncovering distinct phenotypic profiles driven by the holistic lived experience of PwMS. By understanding how different patient profiles aligned with specific 1-year longitudinal autonomy trajectories, this study intended to provide novel and actionable insights. Ultimately, this approach will help Health Care Professionals (HCPs) move beyond standard physical metrics to proactively anticipate and address the unique socio-professional and relational needs of their patients.

## Materials and methods

### FOCAL-MS2 study

**Fig. 1:**
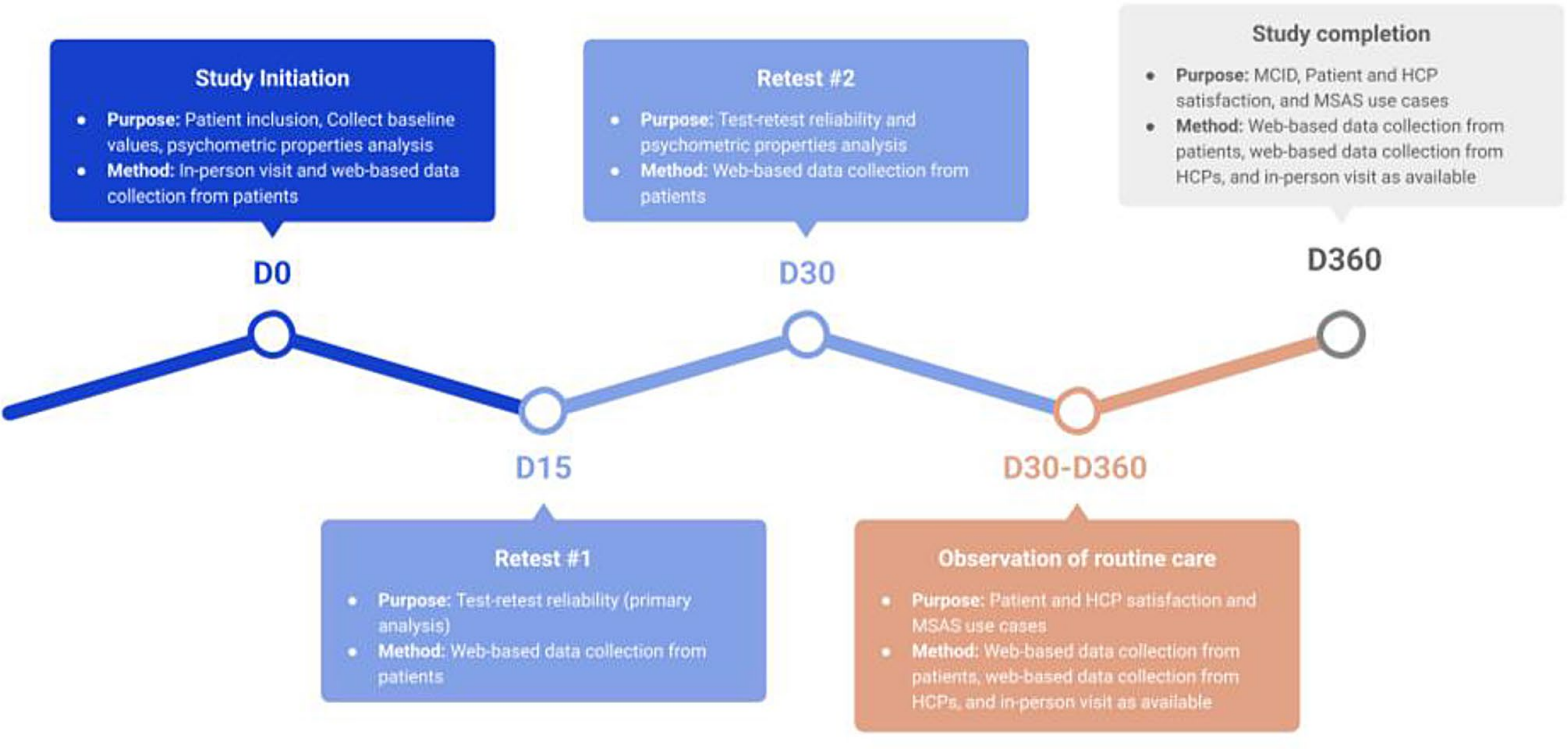
MSAS questionnaire completion timepoints

FOCAL-MS 2 was a non-interventional, prospective, national, multicentric study. Adult patients were eligible for inclusion if they had a confirmed MS diagnosis based on the 2017 McDonald criteria and were affiliated with the French social security system. Between February and April 2024, 199 patients were enrolled and subsequently followed for 12 months in routine care (Fig. 1).

### MSAS questionnaire

The MSAS (Multiple Sclerosis Autonomy Score) comprises 10 dimensions, namely: (D1) Participating in activities with others, (D2) Socio-professional activities, (D3) Controlling the image sent to others, (D4) Consideration at work, (D5) The support of friends, (D6) The consideration of care team, (D7) Support from partner, (D8) Role as a grandparent, (D9) Involvement in an associative group and (D10) Involvement in activities (sports, leisure, travel). Patients first rate the importance of each dimension (0–9 scale or not applicable) and then the Impact of MS (2–4 questions per dimension, 6-item Likert scales). For more details, MSAS questionnaire can be found in supplementary materials number 8. Four dimensions [1, 3–5] apply to all patients, while the remaining six [2, 4, 6–9] are optional, based on patient-specific conditions (e.g., job, partner, being a grandparent). If a dimension’s importance is marked “not applicable”, the corresponding impact questions are skipped. An increase in the MSAS score indicates a decrease in autonomy (scale range from 0:full autonomy to 100:no autonomy). Patients completed the MSAS questionnaire at four structured timepoints: Baseline (D0), Day 15, Day 30, and Day 360. The MSAS is a validated Patient Reported Outcome (PRO) and showed good internal consistency (Cronbach’s alpha = 0.845) and good test-retest reliability (intra-class correlation coefficient = 0.72 at D15 and 0.75 at D30) [2].

### Variable selection for clustering

The variables selected for clustering were chosen based on their medical relevance and reflected dimensions of patient autonomy as defined in the study questionnaire, along with important clinical variables: Sex, Age (in years), Activities (None, Personal, Group), body mass index (BMI) (kg/m2), Time since patient diagnosis (in years), Ability to walk without aid (wheelchair, always aided, occasional aid, functional autonomy), Professional situation (active, on sick leave, retired), Someone helps at home for daily tasks (Yes/No) (more information available in supplementary material number 9).

### Qluster workflow overview

The general clustering process, as outlined in Qluster [7], involves three main steps: pre-processing, data clustering and interpretation of clusters (Figure available in supplementary material 1). Before detailing the specific statistical procedures, the methodology can be summarized in three intuitive steps. First, the workflow simplified complex and diverse patient data by grouping highly correlated characteristics into broader, more manageable dimensions. Second, it used a pattern-recognition algorithm to automatically identify natural groupings (or ‘clusters’) of patients who share similar clinical and sociodemographic profiles. Finally, it rigorously tested these identified groups through repeated simulations to ensure they were mathematically stable and reproducible, confirming that the profiles were real patterns rather than random artifacts of the data, thereby providing a reliable foundation for clinical interpretation.

### Step 1: data pre-processing

The dataset utilized in this study was referred to as “mixed data” because it contains a combination of distinct continuous variables (Age, BMI, and Time since diagnosis) and discrete categorical variables (Sex, Activities, Ability to walk without aid, Professional situation, and Carer at home). To allow the Partitioning Around Medoids (PAM) clustering algorithm (described in step 2) to effectively compute distance metrics, it was necessary to transform this mixed dataset into a unified continuous format. To achieve this, we employed Factor Analysis of Mixed Data (FAMD) [9] using the FactorMineR R package. FAMD was a dimensionality reduction technique specifically designed to handle mixed datasets; it successfully transformed our original diverse set of variables into five new continuous principal dimensions (coordinates). FAMD summary result was available in supplementary material Fig. 2). Importantly, MSAS scores were not used as input variables for generating the clusters. They were utilized to evaluate the autonomy profiles of the resulting clusters.

### Step 2: cluster generation

After dimensionality reduction using FAMD, the Partitioning Around Medoids (PAM) clustering method was applied to the resulting five principal dimensions. PAM was a K-medoid algorithm recognized for its robustness and its ability to minimize a sum of dissimilarities, making it less sensitive to outliers compared to K-means (which relies on squared Euclidean distances). It was also a deterministic algorithm due to its internal medoid initialization procedure, ensuring reproducible clusters. The *pamk()* function from the **FPC R package** V2.2–13 was used.

The optimal number of clusters was determined using the Average Silhouette Width (ASW), an internal validity metric that assesses the compactness within clusters and the separation between clusters [10]. Calinski-Harabasz index [11] and Davies-Bouldin index [12] were also used.

To ensure the robustness and reliability of the clustering results, the stability of the generated clusters was evaluated using the bootstrap method (*N* = 50 iterations), a common approach to perturb the data and assess the consistency of the generated clusters (bootstrap matrix is available in supplementary materials number 3). For each cluster, the Jaccard similarity index [13, 14] was used as the metric to compare the similarity between clusters obtained from bootstrapped samples and the original clusters. A Jaccard index greater than 0.85 indicates high stability.

### Step 3: clustering description

The identified clusters were characterized using lift values [7]. A lift value indicated whether the prevalence of a specific modality (category of a variable) within a cluster was higher, lower, or similar to its prevalence in the overall population. A lift value greater than 1 signified that the modality was more frequent in that cluster, equal to 1 meant the same frequency, and less than 1 means it was less frequent. This method, combined with descriptive statistics, facilitated the interpretation of clusters by highlighting their unique characteristics.

#### Statistical methods

Qualitative data are presented with the number of filled and missing data and, for each variable, the frequency and percentage (among filled data). Quantitative data was reported as arithmetic means and standard deviation. No replacement method of missing data was used in the analysis. While there was no strict sample size requirement for this exploratory analysis, the PAM algorithm was highly robust and well-suited for medium-sized datasets like ours [7].

To evaluate the secondary objective regarding autonomy trajectories over time, within-patient score changes from baseline (Day 0) to Day 360 were calculated for both the global MSAS score and each of the 10 subscale dimensions. To determine the clinical relevance of these variations, the score differences were evaluated against the Minimum Clinically Important Difference (MCID) threshold of 5.7 points, which was established in the primary FOCAL-MS2 study [2] as the smallest change in a score that a patient would perceive as important. Consequently, a score difference over 1 year greater than +5.7 points was interpreted as a clinically significant deterioration (worsening in autonomy), whereas a score difference lower than −5.7 points was considered a clinically significant improvement.

## Results

### Description of the cohort

A total of 199 PwMS were included in the study: N (74%) women, mean age at diagnosis 34.3 ± 9.9 years and 97 (48.7%) have a disease duration > 15 years [2]. Overall, 50 (25.1%) of patients had no leisure at all, 76 (38.2%) were active professionally, 57 (28.6%) were on sick leave and 27 (13.6%) were retired. Regarding mobility, 113 (56.8%) did not require any walking assistance and 19 (9.5%) needed a wheelchair. Finally, 75 (37.7%) of patients had someone regularly helping at home with everyday tasks. Baseline characteristics of the overall cohort can be found in Table 1 below.

**Table 1:** Baseline characteristics of the overall FOCAL-MS2 cohort (*N* = 199)

	Analysis Population (N = 199)
**Gender**, N (%)	
Male	51 (25.6)
Female	148 (74.4)
**Age at diagnosis in years**, mean (SD)	34.3 (9.9)
**Professional situation**, N (%)	
Active	76 (38.2)
On sick leave because of my health	57 (28.6)
Retired	27 (13.6)
Unemployed	23 (11.6)
Other	7 (3.5)
I do not wish to answer	7 (3.5)
Student	2 (1.0)
**Lives alone**, N (%)	57 (28.6)
**Someone regularly helps at home with everyday tasks**, N (%)	75 (37.7)
**Has dependent children**, N (%)	84 (42.2)
**Is a grandparent**, N (%)	31 (15.6)
**Leisure activities**, N (%) [a]	
Personal leisure activities	123 (61.8)
Group leisure activities	64 (32.2)
No leisure activities	50 (25.1)
**Time since MS Diagnosis in years**, mean (SD)	15.4 (10.6)
**Form of MS**, N (%)	
Relapsing remitting	132 (66.3)
Secondarily progressive	44 (22.1)
Primary progressive	23 (11.6)
**Requires assistance to walk**, N (%)	86 (43.2)
**Type or frequency of assistance to walk, N (%) [c]**	
Always requires assistance (cane, walker … )	39 (45.3)
Requires occasional assistance (cane, carer … )	28 (32.6)
In a wheelchair	11 (12.9)
In an electric wheelchair	8 (9.3)

Overall completion rates of the MSAS were high: D0: 100%, *n* = 199; D15: 92.5%, *n* = 184; D30: 91.0%, *n* = 181; D360: 79.4%, *n* = 158).

**Fig. 2:**
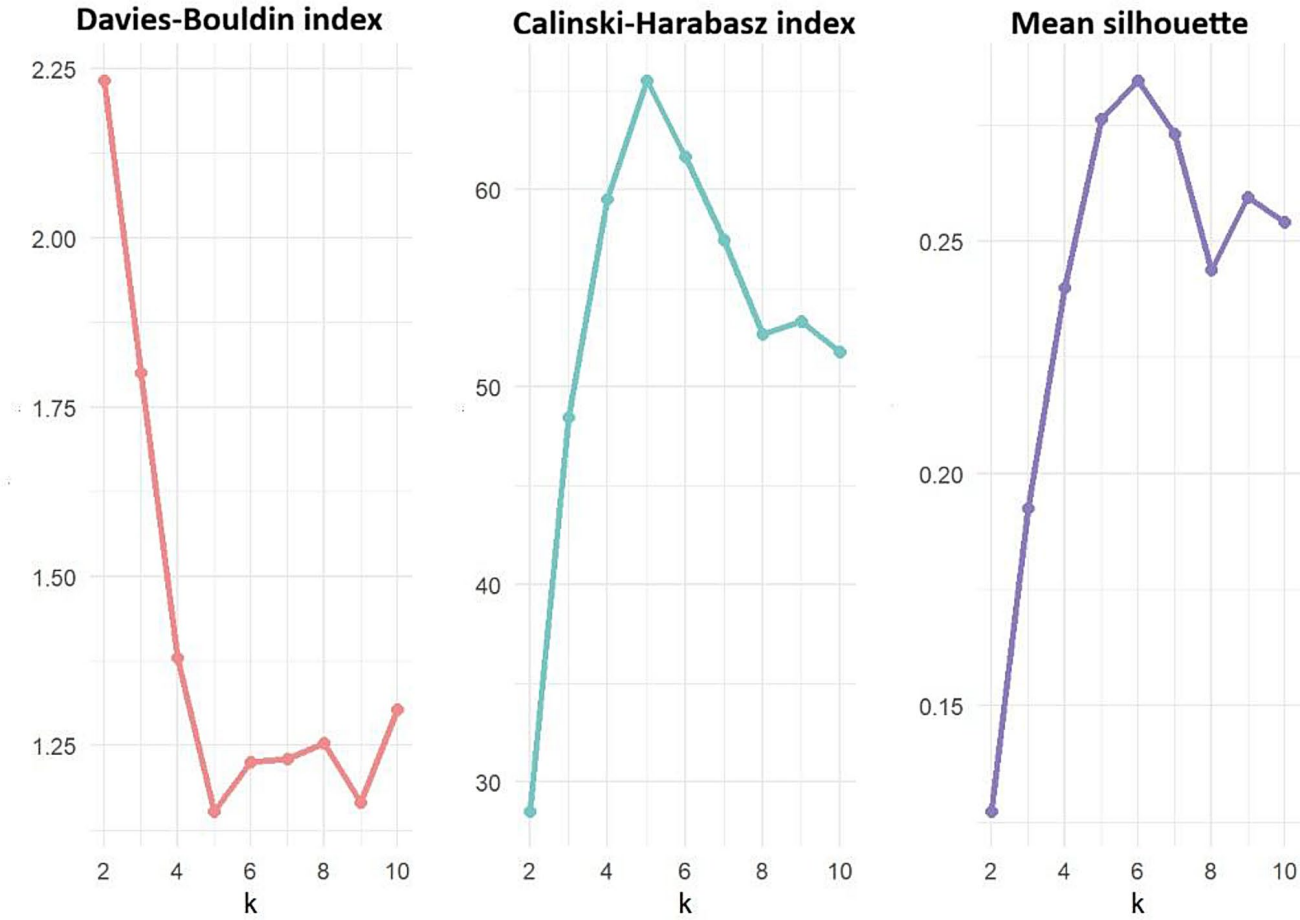
Clustering quality indices

Analysis of the clustering quality indices based on the number of clusters in Figure 2 below led to the selection of k = 6 clusters. The average Silhouette score is 0.28, indicating fairly clear boundaries between clusters, with a Calinski Harabasz index of 61.64 and a Davies-Bouldin index of 1.23. Even if the last 2 indices were higher with 5 clusters, we decided to move forward with 6 clusters as associated Jaccard indices showed better stability than with 5 clusters (data not displayed).

**Fig. 3:**
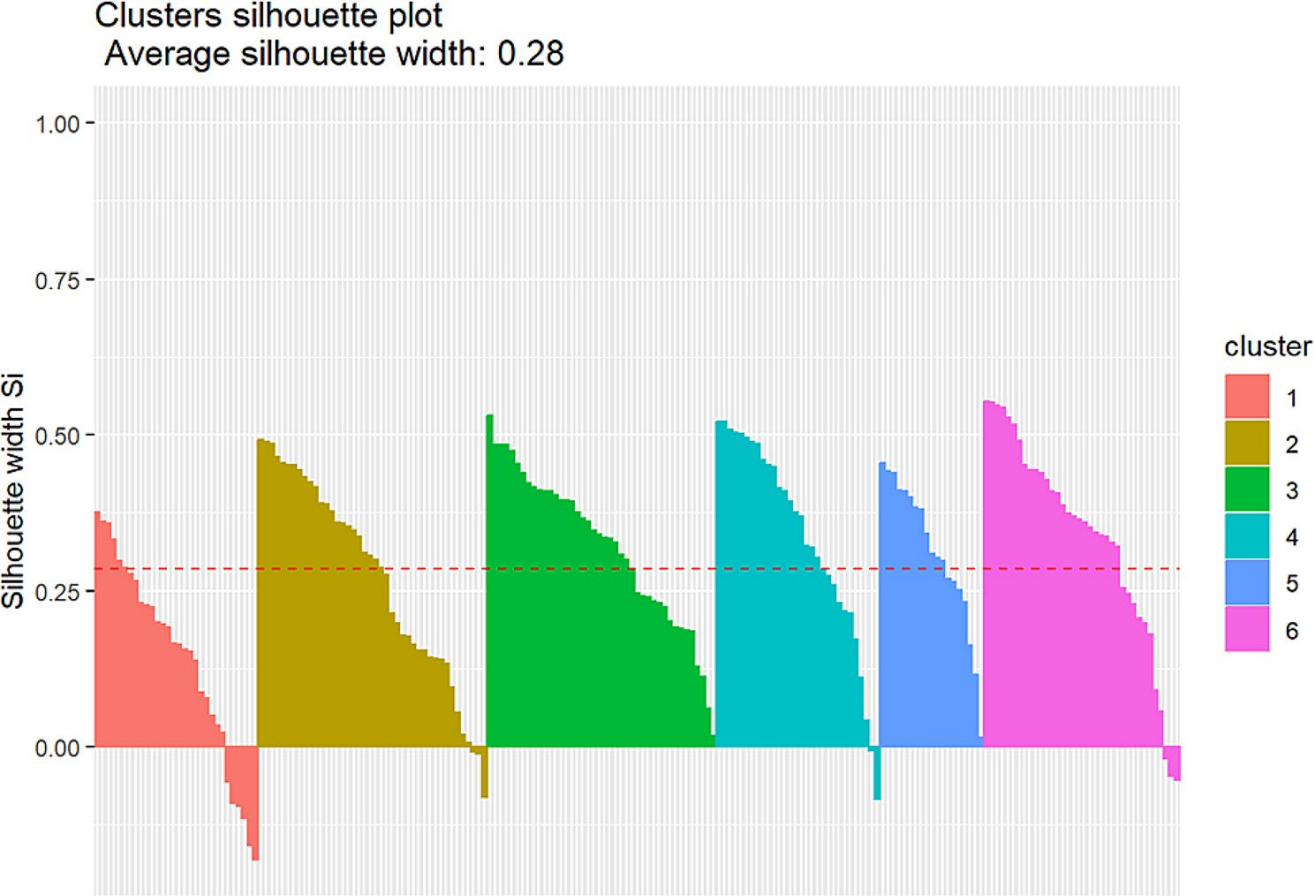
Silhouette width for each patient

The Silhouette Width for each patient further confirmed the good level of intra-cluster cohesion and inter-cluster separability (Fig. 3 below).

The results demonstrated high stability for all clusters, indicating that the identified profiles were robust and not merely artifacts of the specific dataset sample. The mean Jaccard indices across all clusters were consistently high, ranging from 0.90 for cluster 1 to 0.98 for cluster 6 (see Figure 6 in supplementary materials for details of Jaccard index per cluster).

### Description of clusters

The number of patients per cluster ranged from 19 to 42 patients. The six clusters are described below, highlighting their defining characteristics based on the lift values and prevalence data. We used a lift value greater than 1.25, indicating an overrepresentation of the modality within the cluster relative to its prevalence in the overall cohort, to describe below the main characteristics of each cluster.

Cluster 1 (*n* = 30): This group was predominantly composed of patients aged between 50 and 60 years (lift = 1.78) without personal activities (lift = 2.53). They were more frequently on sick leave (lift = 1.86), required a carer at home (lift = 1.86), and had the highest lift for using a wheelchair for mobility (lift = 3.14). Their MS duration was typically greater than 15 years (lift = 1.30).

Cluster 2 (*n* = 42): Characterized primarily by male patients (lift = 1.77) aged between 40 and 50 years (lift = 3.07). They often required occasional walking assistance (lift = 1.86), and their time since diagnosis was generally less than 5 years (lift = 1.39).

Cluster 3 (*n* = 42): This cluster consisted of patients predominantly aged between 50 and 60 years (lift = 2.83) who were highly engaged in personal activities (lift = 1.58). They also required a wheelchair for mobility (lift = 1.50) and had a time since diagnosis typically greater than 15 years (lift = 1.32).

Cluster 4 (*n* = 30): This group was strongly defined by retired patients (lift = 6.14) aged 60 years or older (lift = 5.34). They benefited from a carer at home (lift = 1.68) and frequently required constant walking aid (lift = 2.55). Their MS duration was typically greater than 15 years (lift = 1.91).

Cluster 5 (*n* = 19): This smaller cluster comprises younger patients under 40 years old (lift = 2.49) who reported having no activities (lift = 3.98). They did not require a carer at home (lift = 1.52), maintain functional autonomy for walking (lift = 1.48), and had a time since diagnosis of less than 5 years (lift = 2.81).

Cluster 6 (*n* = 36): Primarily composed of women (lift = 1.27) under 40 years old (lift = 3.42) who were professionally active (lift = 1.89). Like Cluster 5, they did not require a carer at home (lift = 1.38), maintained functional autonomy for walking (lift = 1.57), and their time since diagnosis was typically less than 5 years (lift = 1.75) or between 5 and 15 years (lift = 1.36).

Boxplot of time since MS diagnosis, body mass index and age distribution per cluster can be found in supplementary figures 4, 5 and 6.

**Fig. 4:**
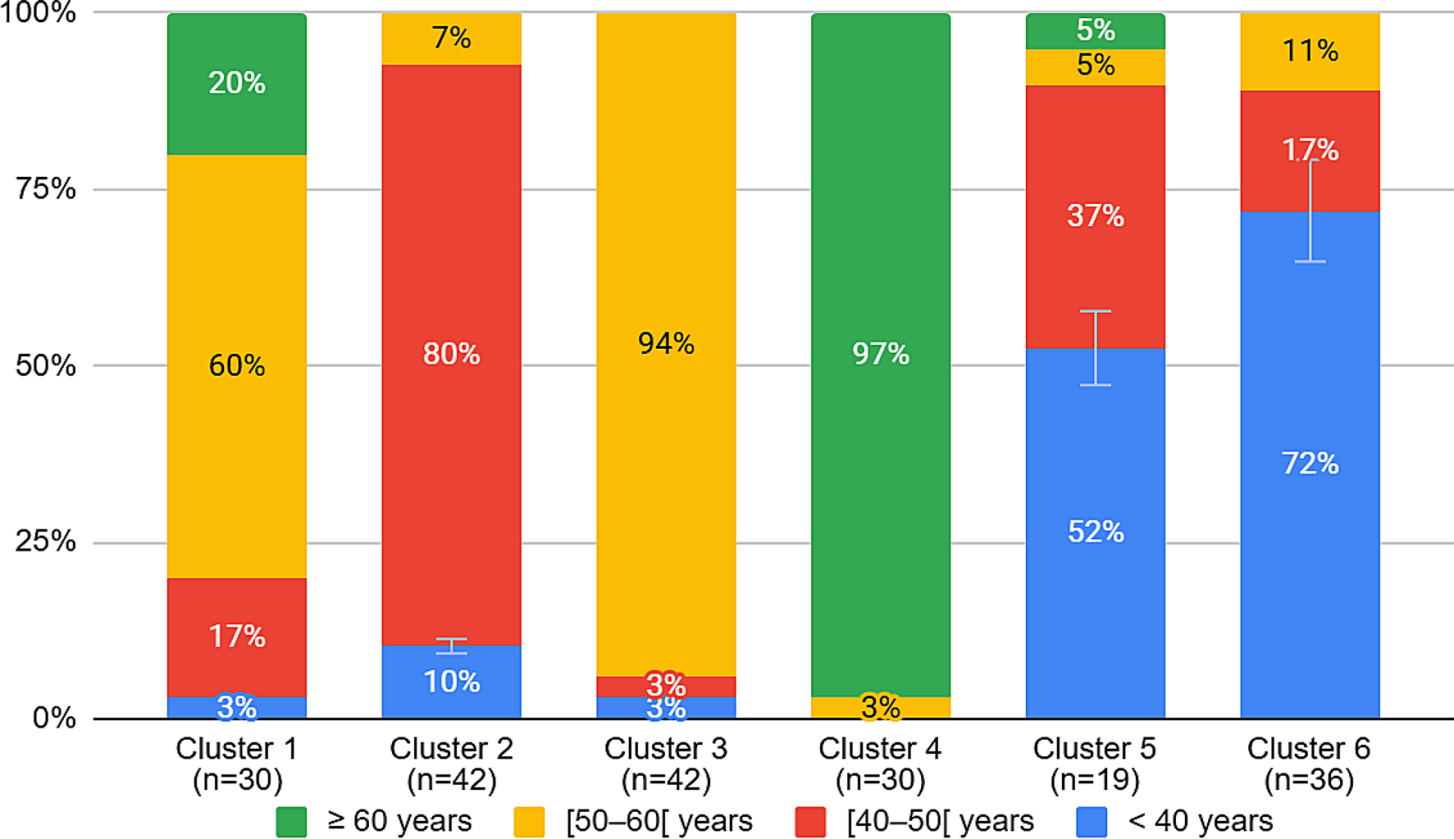
Age categories per cluster

Figure 4 below describes age categories per cluster.

Clusters were described in Table 2 according to variables selected.

**Table 2:** Characteristics of the included population at baseline according to clusters

Variable	Category	Cluster 1 (*n* = 30)	Cluster 2 (*n* = 42)	Cluster 3 (*n* = 42)	Cluster 4 (*n* = 30)	Cluster 5 (*n* = 19)	Cluster 6 (*n* = 36)	Analysis Population (*N* = 199)	Lift C1	Lift C2	Lift C3	Lift C4	Lift C5	Lift C6
Sex, N (%)	Male	7 (23.3%)	19 (45.2%)	8 (19.0%)	9 (30.0%)	6 (31.6%)	2 (5.6%)	51 (25.6%)	0.91	1.77	0.74	1.17	1.23	0.22
Sex, N (%)	Female	23 (76.7%)	23 (54.8%)	34 (81.0%)	21 (70.0%)	13 (68.4%)	34 (94.4%)	148 (74.4%)	1.03	0.74	1.09	0.94	0.92	1.27
Age (years), N (%)	<40 years	1 (3.3%)	4 (9.5%)	1 (2.4%)	0	10 (52.6%)	26 (72.2%)	42 (21.1%)	0.16	0.45	0.11	-	2.49	3.42
Age (years), N (%)	[40–50[years	5 (16.7%)	35 (83.3%)	1 (2.4%)	0	7 (36.8%)	6 (16.7%)	54 (27.1%)	0.61	3.07	0.09	-	1.36	0.61
Age (years), N (%)	[50–60[years	18 (60.0%)	3 (7.1%)	40 (95.2%)	1 (3.3%)	1 (5.3%)	4 (11.1%)	67 (33.7%)	1.78	0.21	2.83	0.1	0.16	0.33
Age (years), N (%)	≥60 years	6 (20.0%)	0	0	29 (96.7%)	1 (5.3%)	0	36 (18.1%)	1.11	-	-	5.34	0.29	-
Personal activities, N (%)	Yes	1 (3.3%)	39 (92.9%)	41 (97.6%)	18 (60.0%)	0	24 (66.7%)	123 (61.8%)	0.05	1.5	1.58	0.97	-	1.08
Personal activities, N (%)	No	29 (96.7%)	3 (7.1%)	1 (2.4%)	12 (40.0%)	19 (100.0%)	12 (33.3%)	76 (38.2%)	2.53	0.19	0.06	1.05	2.62	0.87
Group activities, N (%)	Yes	2 (6.7%)	9 (21.4%)	9 (21.4%)	15 (50.0%)	0	29 (80.6%)	64 (32.2%)	0.21	0.67	0.67	1.55	-	2.5
Group activities, N (%)	No	28 (93.3%)	33 (78.6%)	33 (78.6%)	15 (50.0%)	19 (100.0%)	7 (19.4%)	135 (67.8%)	1.38	1.16	1.16	0.74	1.47	0.29
No activities, N (%)	Yes	27 (90.0%)	0	0	4 (13.3%)	19 (100.0%)	0	50 (25.1%)	3.58	-	-	0.53	3.98	-
No activities, N (%)	No	3 (10.0%)	42 (100.0%)	42 (100.0%)	26 (86.7%)	0	36 (100.0%)	149 (74.9%)	0.13	1.34	1.34	1.16	-	1.34
Body Mass Index, N (%)	<18.5	3 (10.0%)	1 (2.4%)	3 (7.1%)	0	0	5 (13.9%)	12 (6.0%)	1.66	0.39	1.18	-	-	2.3
Body Mass Index, N (%)	[18.5–25[	9 (30.0%)	24 (57.1%)	18 (42.9%)	17 (56.7%)	12 (63.2%)	19 (52.8%)	99 (49.7%)	0.6	1.15	0.86	1.14	1.27	1.06
Body Mass Index, N (%)	[25–30[	11 (36.7%)	11 (26.2%)	13 (31.0%)	10 (33.3%)	4 (21.1%)	11 (30.6%)	60 (30.2%)	1.22	0.87	1.03	1.11	0.7	1.01
Body Mass Index, N (%)	≥30	7 (23.3%)	6 (14.3%)	8 (19.0%)	3 (10.0%)	3 (15.8%)	1 (2.8%)	28 (14.1%)	1.66	1.02	1.35	0.71	1.12	0.2
Time since diagnosis, N (%)	<5 years	2 (6.7%)	12 (28.6%)	3 (7.1%)	0	11 (57.9%)	13 (36.1%)	41 (20.6%)	0.32	1.39	0.35	-	2.81	1.75
Time since diagnosis, N (%)	[5–15] years	9 (30.0%)	16 (38.1%)	12 (28.6%)	2 (6.7%)	7 (36.8%)	15 (41.7%)	61 (30.7%)	0.98	1.24	0.93	0.22	1.2	1.36
Time since diagnosis, N (%)	>15 years	19 (63.3%)	14 (33.3%)	27 (64.3%)	28 (93.3%)	1 (5.3%)	8 (22.2%)	97 (48.7%)	1.3	0.68	1.32	1.91	0.11	0.46
Ability to walk without aid, N (%)	In a wheelchair	9 (30.0%)	0	6 (14.3%)	4 (13.3%)	0	0	19 (9.5%)	3.14	-	1.5	1.4	-	-
Ability to walk without aid, N (%)	always aided	9 (30.0%)	3 (7.1%)	9 (21.4%)	15 (50.0%)	1 (5.3%)	2 (5.6%)	39 (19.6%)	1.53	0.36	1.09	2.55	0.27	0.28
Ability to walk without aid, N (%)	occasional aid	7 (23.3%)	11 (26.2%)	2 (4.8%)	4 (13.3%)	2 (10.5%)	2 (5.6%)	28 (14.1%)	1.66	1.86	0.34	0.95	0.75	0.39
Ability to walk without aid, N (%)	functional autonomy	5 (16.7%)	28 (66.7%)	25 (59.5%)	7 (23.3%)	16 (84.2%)	32 (88.9%)	113 (56.8%)	0.29	1.17	1.05	0.41	1.48	1.57
Professional situation, N (%)	active	6 (20.0%)	16 (38.1%)	18 (42.9%)	1 (3.3%)	9 (47.4%)	26 (72.2%)	76 (38.2%)	0.52	1.0	1.12	0.09	1.24	1.89
Professional situation, N (%)	on sick leave	16 (53.3%)	8 (19.0%)	19 (45.2%)	3 (10.0%)	5 (26.3%)	6 (16.7%)	57 (28.6%)	1.86	0.66	1.58	0.35	0.92	0.58
Professional situation, N (%)	Retired	2 (6.7%)	0	0	25 (83.3%)	0	0	27 (13.6%)	0.49	-	-	6.14	-	-
Professional situation, N (%)	Other	6 (20.0%)	18 (42.9%)	5 (11.9%)	1 (3.3%)	5 (26.3%)	4 (11.1%)	39 (19.6%)	1.02	2.19	0.61	0.17	1.34	0.57
Carer at home, N (%)	Yes	21 (70.0%)	13 (31.0%)	16 (38.1%)	19 (63.3%)	1 (5.3%)	5 (13.9%)	75 (37.7%)	1.86	0.82	1.01	1.68	0.14	0.37
Carer at home, N (%)	No	9 (30.0%)	29 (69.0%)	26 (61.9%)	11 (36.7%)	18 (94.7%)	31 (86.1%)	124 (62.3%)	0.48	1.11	0.99	0.59	1.52	1.38

### Baseline global autonomy score

Cluster 4 (*n* = 30) had the highest global means score of 41.8 (14.0). Given that an increase in the MSAS score indicated a decrease in autonomy, this was the most significantly impacted group at baseline. Cluster 6 (*n* = 36) had the lowest global mean score of 28.6 (12.0) which corresponds to the highest level of initial autonomy among all groups. MSAS scores at baseline according to clusters are described in Table 3.

**Table 3:** MSAS scores at baseline according to clusters

Dimension description	Cluster 1 (*n* = 30)	Cluster 2 (*n* = 42)	Cluster 3 (*n* = 42)	Cluster 4 (*n* = 30)	Cluster 5 (*n* = 19)	Cluster 6 (*n* = 36)	Analysis Population (*N* = 199)
MSAS global score at baseline mean (SD)	35.8 (13.5)	38.7 (12.1)	34.4 (12.9)	41.8 (14.0)	35.4 (12.7)	28.6 (12.0)	35.7 (13.3)
D1: Participating in activities with others	54.8 (16.2)	41.1 (23.3)	39.5 (22.7)	54.5 (24.0)	40.5 (20.7)	26.2 (20.2)	42.1 (23.4)
D2: Socio-professional activities	30.0 (31.4)	35.5 (28.4)	31.4 (22.8)	33.3 (23.7)	28.8 (31.9)	31.2 (20.6)	31.9 (25.7)
D3: Controlling the image sent to others	26.4 (21.0)	30.6 (27.2)	23.5 (20.5)	24.4 (22.0)	28.4 (22.6)	19.4 (17.3)	25.3 (22.1)
D4: Consideration at work	45.0 (32.9)	45.0 (22.2)	40.0 (26.8)	58.7 (37.5)	48.4 (23.3)	33.8 (22.1)	42.0 (25.6)
D5: The support of friends	35.3 (22.4)	51.3 (22.4)	38.6 (19.7)	42.9 (20.6)	35.8 (29.1)	42.8 (25.2)	41.9 (23.2)
D6: The consideration of care team	21.7 (22.9)	30.0 (26.4)	26.4 (24.6)	29.7 (26.1)	25.3 (15.4)	25.0 (17.3)	26.6 (23.0)
D7: Support from partner	26.8 (22.8)	30.3 (22.9)	33.6 (26.7)	36.4 (28.0)	36.4 (24.7)	37.2 (21.4)	33.3 (24.3)
D8: Role as a grandparent	35.0 (49.5)	100.0 (NA)	32.5 (18.3)	53.5 (31.0)	30.0 (NA)	NA	47.2 (30.5)
D9 : Involvement in associative group	47.5 (45.6)	60.9 (30.6)	61.3 (33.2)	58.6 (38.0)	56.7 (51.3)	33.5 (29.9)	53.1 (35.5)
D10: Involvement in activities (sports, leisure, travel)	47.1 (35.6)	28.7 (28.4)	26.2 (22.1)	40.5 (33.1)	40.0 (20.0)	14.1 (20.2)	28.8 (28.2)

### Baseline most impacted dimension per cluster

Cluster 1 (middle-aged, on sick leave): These patients were most impacted in their social and leisure lives at baseline, showing the highest scores in Participating in activities with others (D1) (mean score: 54.8), Involvement in an associative group (D9) (47.5), and Involvement in activities (D10) (47.1).

Cluster 2 (younger, with occasional walking assistance): This group demonstrated a profound baseline impact regarding their Involvement in an associative group (D9) (60.9) and The support of friends (D5) (51.3).

Cluster 3 (middle-aged, with personal activities): The autonomy of these patients was most significantly restricted in Involvement in an associative group (D9) (61.3), followed by Consideration at work (D4) (40.0).

Cluster 4 (elderly, with leisure): Corresponding to their high disability status, these patients exhibited prominent baseline impacts across multiple dimensions, most notably Consideration at work (D4) (58.7), Involvement in an associative group (D9) (58.6), Participating in activities with others (D1) (54.5), and Role as a grandparent (D8) (53.5).

Cluster 5 (younger, no activities): The highest baseline impacts for this cohort were observed in social and professional spheres, specifically Involvement in an associative group (D9) (56.7) and Consideration at work (D4) (48.4).

Cluster 6 (younger, professionally active women): While maintaining the best overall global score, this group experienced their highest baseline autonomy impact in interpersonal relationships, specifically regarding The support of friends (D5) (42.8) and Support from partner (D7) (37.2).

### Cluster’s evolution of autonomy score

The analysis of the global autonomy mean score revealed distinct trajectories for each cluster, providing insights into the disease’s progression and impact on daily life. We also explored the clusters through the different dimensions of the questionnaire, using the MCID threshold of 5.7 determined in the study [2], this threshold representing the smallest change in a score that a patient would perceive as important. This enabled us to identify areas in which the patient’s autonomy significantly deteriorated (score difference over 1 year >+5.7) or improved (score difference over 1 year < −5.7).

**Fig. 5:**
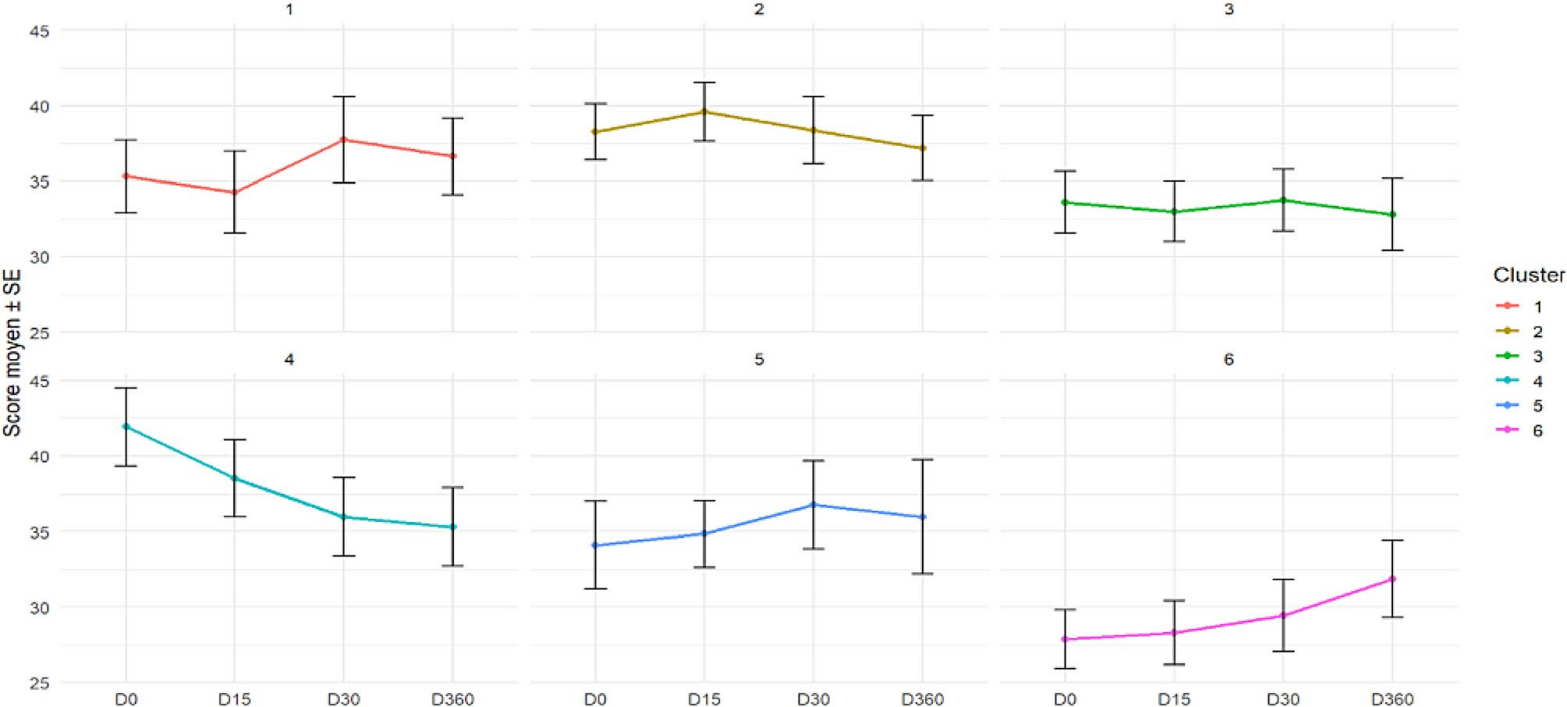
Global score evolution over the study period per cluster

**Clusters 1, 2, 3 and 5:** The evolution of autonomy global scores over one year remained largely stable for these groups, whereas cluster 4 had score decreasing over time and cluster 6 global score increasing over time. Figure 5 below describes the evolution of the MSAS global mean score per cluster (mean score per dimension are available in supplementary materials Figure 7).

**Cluster 1** (middle-aged, on sick leave) patients experienced a significant mean improvement in their autonomy in dimension 1 (Activities with others) with a mean score difference of −7.6 (±22.6) but a significant mean worsening in dimension 6 (consideration of care team) with +6.4 (±18.6).

**Cluster 2** (younger, with occasional walking assistance) showed its most notable improvement in autonomy in dimension 9 (Family support) with a mean score difference of −11.1 (±19.2) but a significant worsening in dimension 5 (The support of friends) with a mean score difference of +10.9 (±24.9).

**Cluster 3** (middle-aged, with personal activities) patients experienced significant worsening in autonomy in dimension 4 (Consideration at work) with +8.6 (±12.7) and dimension 8 (Role as grandparent) with +10.0 (±14.1).

**Cluster 4** (retired with leisure) was characterized by a significant improvement in dimension 1 (Activities with others) with −9.8 (±23.5), while showing a clinical worsening (decrease in autonomy) in dimension 9 (Family support) with +12.5 (±35.4).

**Cluster 5** (younger, without activities) showed a notable worsening in autonomy in dimension 3 (Controlling the image sent to others) with a mean score difference of +9.2 (±23.4) and in dimension 5 (The support of friends) with a mean score difference of +23.8 (±27.2).

**Cluster 6** (young, professionally active women) patients presented an improvement in autonomy in dimension 9 (Family support) with −8.3 (±14.4), but showed a significant worsening in dimensions 3, 4, 6, 7 and 8.

All clusters showed an important worsening in dimension 10 (Involvement in activities). Results are summarized in Table 2 per dimension and per cluster.

Table 4 describes the one-year change from baseline score per dimension.

**Table 4:**
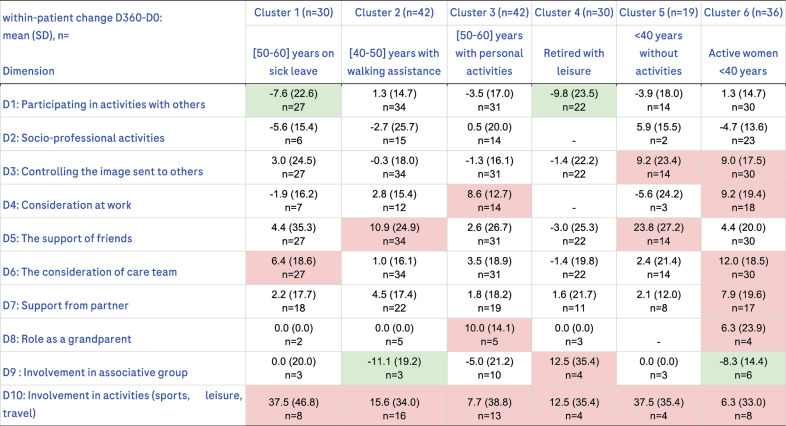
One year change from baseline score per dimension

Footnote: Highlighted cells in red are dimensions with a clinically significant loss of autonomy in 1 year. Highlighted cells in green are dimensions with a clinically significant improvement of autonomy in 1 year

## Discussion and conclusion

### Discussion

In health data analysis, clustering methods are a primary tool for finding pockets of homogeneity within a heterogeneous population, to uncover different disease phenotypes, stages of a disease, or variations in disease outcomes [15]. This study successfully applied an unsupervised clustering approach to identify six distinct profiles among People with MS (PwMS). By clustering patients based on clinical and sociodemographic features without inputting their MSAS scores, we demonstrated that these profiles naturally align with distinct trajectories of perceived autonomy over a 12-month period. For Health Care Professionals (HCPs), these findings offer critical actionable insights. For instance, Cluster 6 (young, professionally active women) presented high physical autonomy but suffered a significant 1-year deterioration in perceived friend support and self-image. This highlights a hidden psychosocial vulnerability that routine clinical metrics (like EDSS) might miss, suggesting an acute need for proactive psychological support. Conversely, Cluster 1 (middle-aged, on sick leave) experienced rapid declines in social activities, indicating a need for early social interventions to prevent isolation. Paradoxically, the most severely disabled group, Cluster 4 (elderly, retired), showed overall improvement in autonomy, which may reflect psychological adaptation or the stabilization of home-care routines. The analysis of the evolution of the change from baseline by subscale scores showed which dimension was most significantly impacted for each cluster and allowed to identify important trends in all health-related quality of life dimensions.

Nowadays, MS management is mainly based on monitoring clinical and radiological results: relapses, Expanded Disability Status Scale (EDSS), lesions observed on magnetic resonance imaging (MRI). The complexity of the disease, the difficulty in determining treatment, and the wide range of symptoms require a comprehensive approach to patients with MS. It is therefore necessary to expand the care of PwMS in order to fully recognize the burden of the disease on daily activities, which leads to a certain loss of independence.

The MSAS provides a complementary, comprehensive assessment of autonomy by evaluating the impact of MS on 10 dimensions. Unlike traditional PROs, which often focus on physical symptoms or functional limitations, the MSAS emphasizes the subjective perspective of the patient, considering what matters most to them in daily life.

The MSAS questionnaire was widely completed throughout the study, leading to high retention rates, and therefore precluded any imputation of missing data.

#### Strengths and limitations

The strengths of this study include its prospective design and the use of the Qluster methodology, which ensured highly stable and reproducible clusters: the prospective design collected structured and longitudinal data over a 12-month period in a real-world setting, and the application of the robust Qluster methodology—combining Factor Analysis of Mixed Data (FAMD) and Partitioning Around Medoids (PAM)—ensured the identification of highly stable and mathematically reproducible patient clusters, mitigating the subjectivity often associated with unsupervised machine learning.

Our cohort is also representative of the general multiple sclerosis (MS) population [16]. Specifically, the male/female ratio in our study (2.9) is comparable to that observed in the general MS population in France, with approximately 3 women for every 1 man [1].

However, limitations include the relatively small sample size per cluster and the limitations inherent to factor analysis, which treats ordinal variables as categorical. Furthermore, while the ASW metric ensures statistical separation, clinical judgment remains essential to interpret these profiles. A small number of patients per cluster were identified due to limited study sample size. However, methods applied were developed for such small sample sizes and clusters showed a good stability.

Four timepoints were available throughout the study, which limits the findings of the evolution of the autonomy scores. Further studies should include a longer timeframe of evaluation of the MSAS questionnaire.

The Qluster workflow acknowledges that no single clustering method is universally optimal for all problems. Drawbacks of Factor Analysis: As a first step, factor analysis using the current packages cannot inherently handle the ordinal nature of variables (treating them as categorical or continuous). Optimization based solely on statistical criteria like Average Silhouette Width (ASW) may not always align with the clinical relevance or usefulness of the generated clusters [7].

### Conclusion

In conclusion, our study identified six unique phenotypes of PwMS characterized by distinct baseline features and 1-year autonomy trajectories. Rather than a homogeneous decline, autonomy in MS evolves dynamically depending on age, professional status, and social support. These findings underline the necessity for clinical management to move beyond monitoring purely physical symptoms. By integrating tools like the MSAS, HCPs can deliver truly personalized care, addressing the specific socio-professional and psychosocial dimensions of autonomy unique to each patient’s profile.

These findings highlight that clinical management must move beyond monitoring physical symptoms like relapses or MRI lesions to address the specific socio-professional and familial dimensions of autonomy unique to each patient profile. The analysis of autonomy score evolution within each cluster provides a nuanced understanding of the disease’s heterogeneity, reinforcing medical knowledge and offering a practical solution for data scientists to uncover clinically relevant patient profiles.

## Electronic supplementary material

Below is the link to the electronic supplementary material.

Supplementary Materials 1

Supplementary Material 2

Supplementary Material 3

Supplementary Material 4

## Data Availability

Qualified researchers may request access to individual patient level clinical data through data_sharing.france@roche.com. As the study has been conducted in France, any request for data sharing will need to comply with French regulations and data privacy principles.

## Abbreviations

ASW: Average Silhouette Width
BMI: Body Mass Index
FAMD: Factor Analysis of Mixed Data
HCP: Health Care Professional
MCID: Minimal Clinically Important Difference
MS: Multiple Sclerosis
MSAS: Multiple Sclerosis Autonomy Scale
PAM: Partitioning Around Medoids
PwMS: Patients with Multiple Sclerosis
