# Supplementary material for: A clustering-based approach to characterize autonomy profiles among multiple sclerosis patients: an application of the Qluster method in the FOCAL-MS2 study
Source: J Patient Rep Outcomes. 2026 May 25;10:125. doi: 10.1186/s41687-026-01074-5 (PMC13396066; doi:10.1186/s41687-026-01074-5)
Supplement: Supplementary file 1 — Supplementary Materials 1 [file 41687_2026_1074_MOESM1_ESM.docx]

**Supplementary materials:**

Suppl. Material 1: Qluster clustering workflow applied to this research


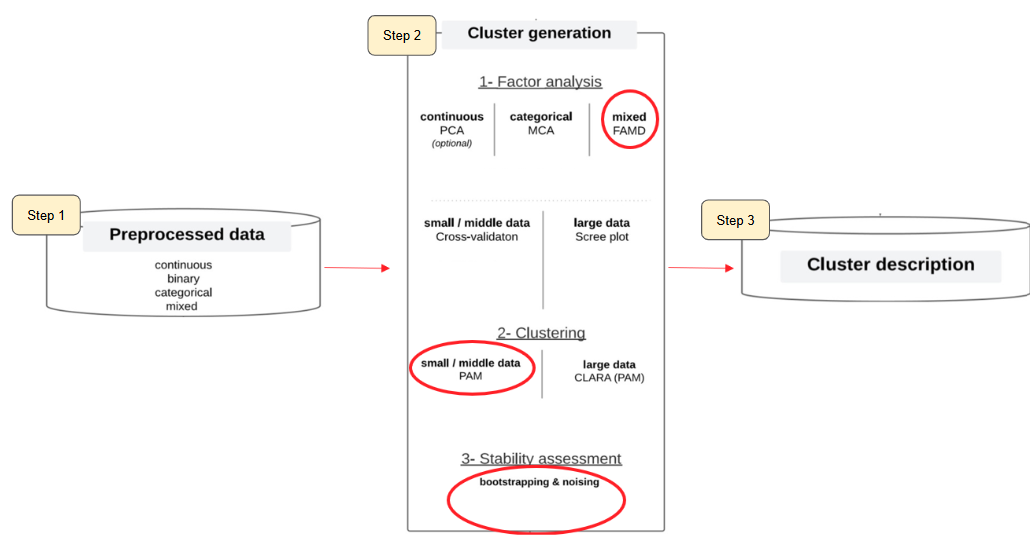


*Footnote: in red are highlighted the chosen steps from the Qqluster algorithm*

Suppl. material 2: FAMD


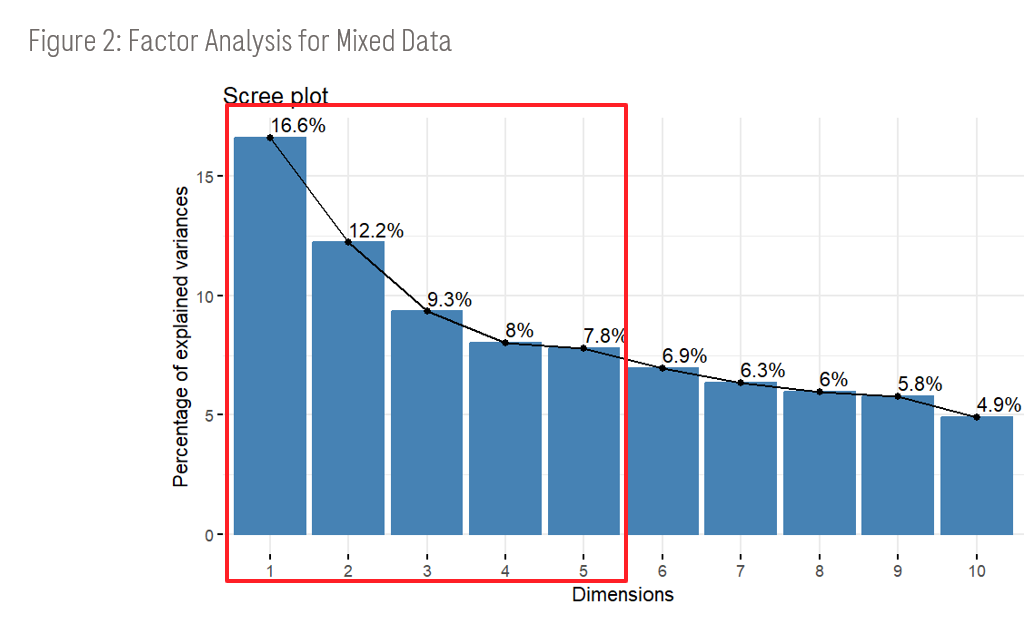


Note: The application of FAMD to the study data successfully explained 54% of the variance with the first five components, thereby retaining more than half of the structural information present in the dataset.

Suppl. Material 3: bootstrap matrix


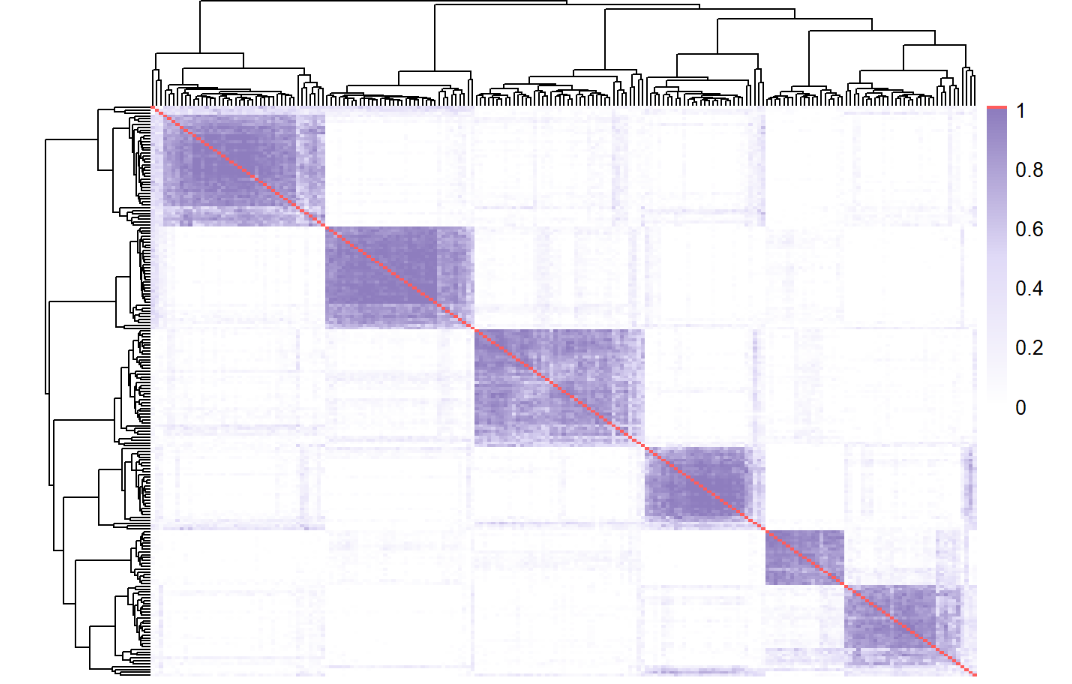


Suppl. Material 4: boxplot of MS duration per cluster


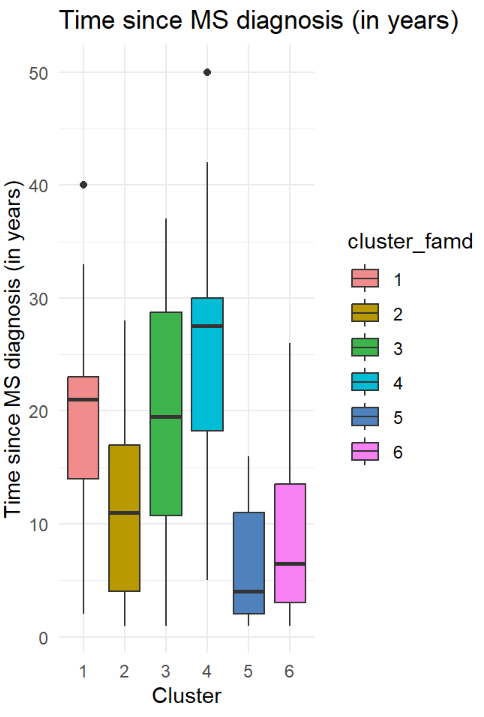


Footnote:

Cluster 1 (middle-aged, on sick leave)

Cluster 2 (younger, with occasional walking assistance)

Cluster 3 (middle-aged, with personal activities)

Cluster 4 (elderly, retired with leisure)

Cluster 5 (younger, without activities)

Cluster 6 (young, professionally active women)

Suppl. Material 5: boxplot of Body Mass Index (BMI) per cluster


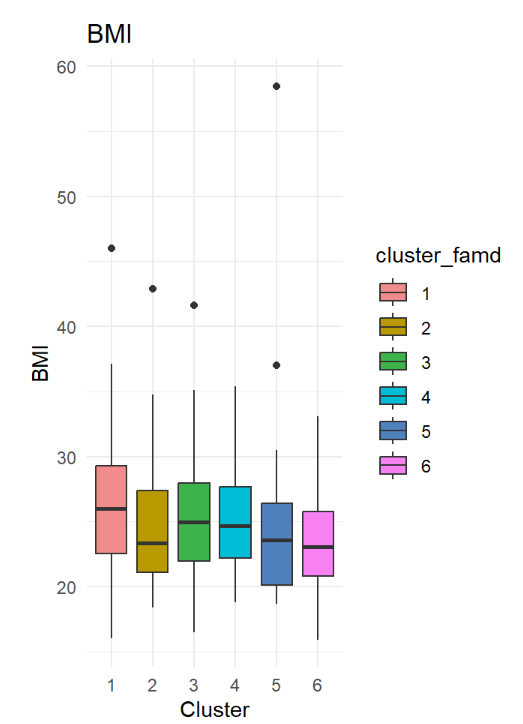


Footnote:

Cluster 1 (middle-aged, on sick leave)

Cluster 2 (younger, with occasional walking assistance)

Cluster 3 (middle-aged, with personal activities)

Cluster 4 (elderly, retired with leisure)

Cluster 5 (younger, without activities)

Cluster 6 (young, professionally active women)

Suppl. Material 6: clusters stability evaluated by the Jaccard index


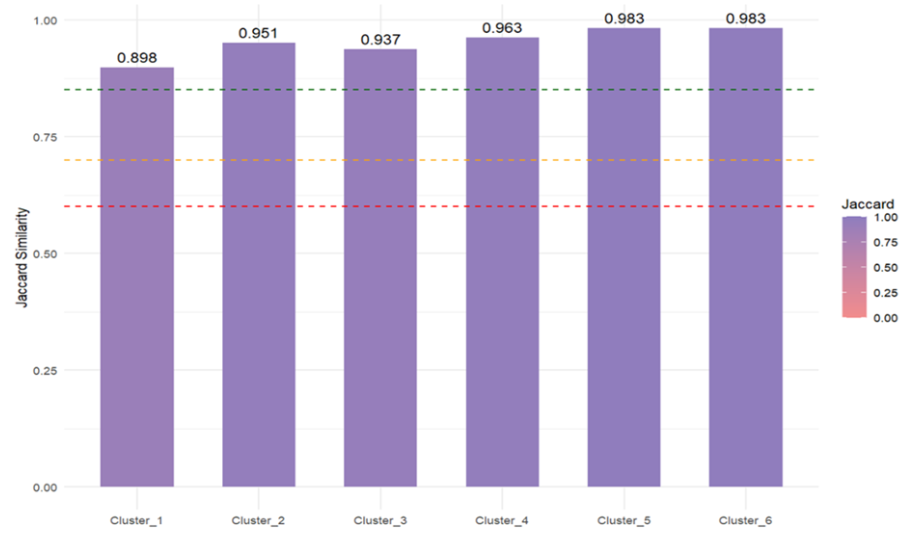


Footnote:

Cluster 1 (middle-aged, on sick leave)

Cluster 2 (younger, with occasional walking assistance)

Cluster 3 (middle-aged, with personal activities)

Cluster 4 (elderly, retired with leisure)

Cluster 5 (younger, without activities)

Cluster 6 (young, professionally active women)

Suppl. Material 7: dimension mean score evolution over the study period per cluster

| 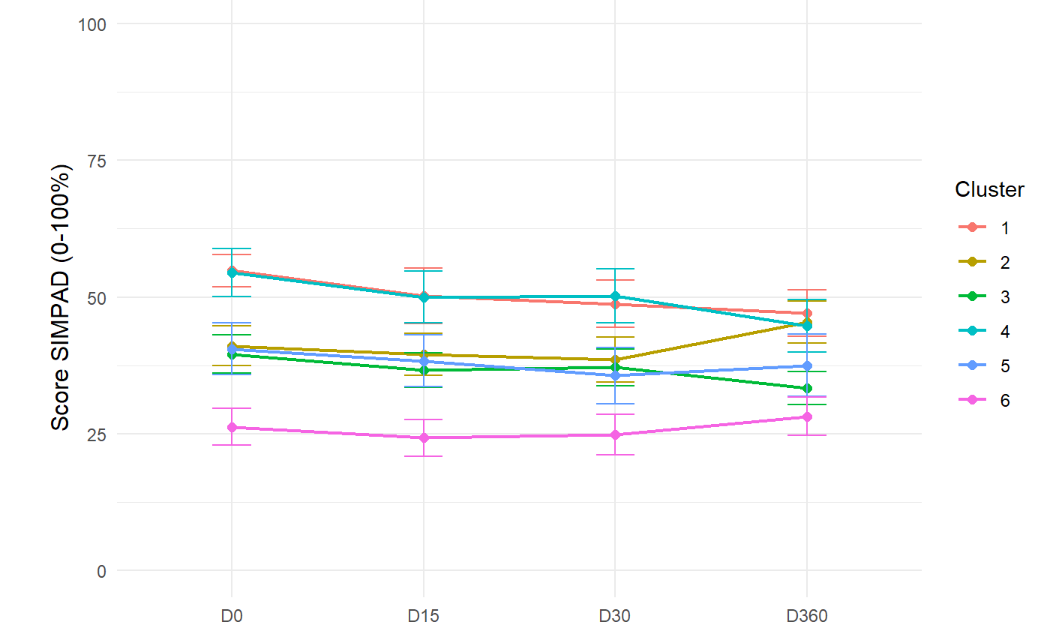  Dimension 1: Participating in activities with others | 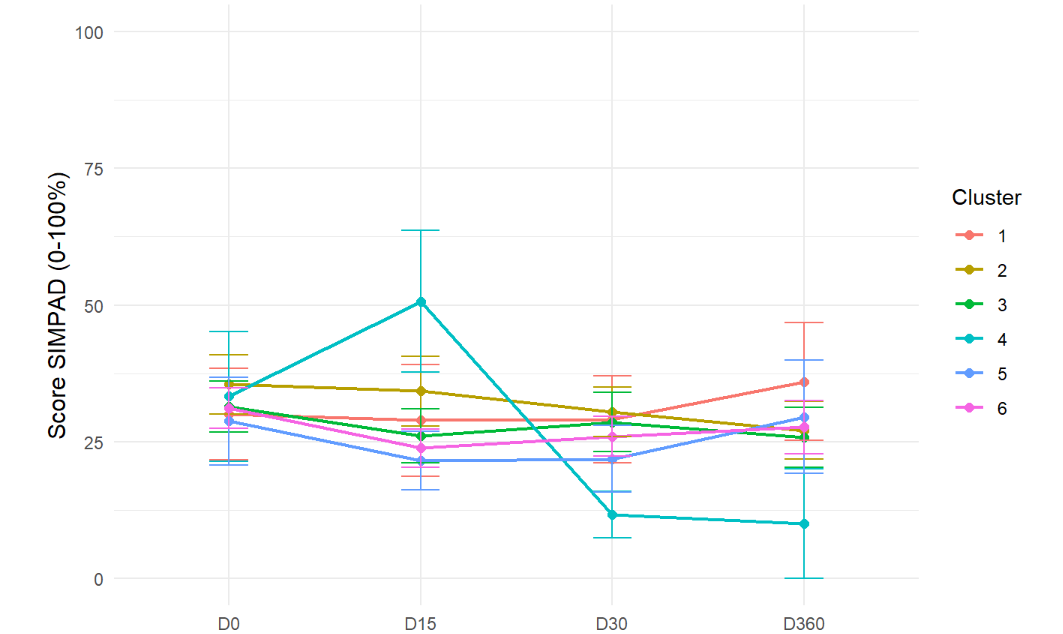  Dimension 2: Socio-professional activities |
| --- | --- |
| 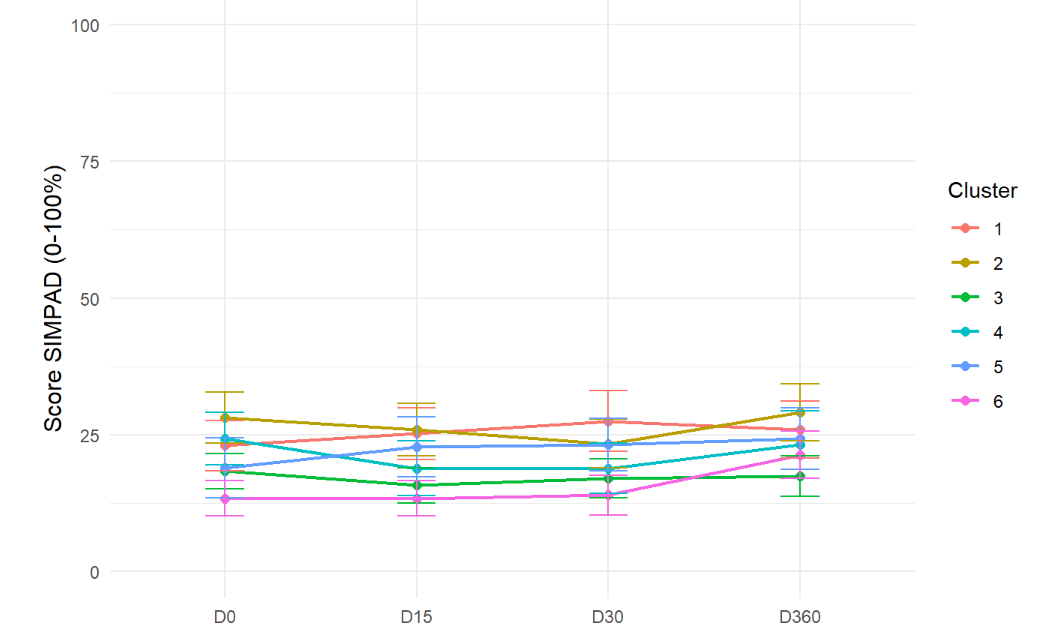  Dimension 3: Controlling the image sent to others | 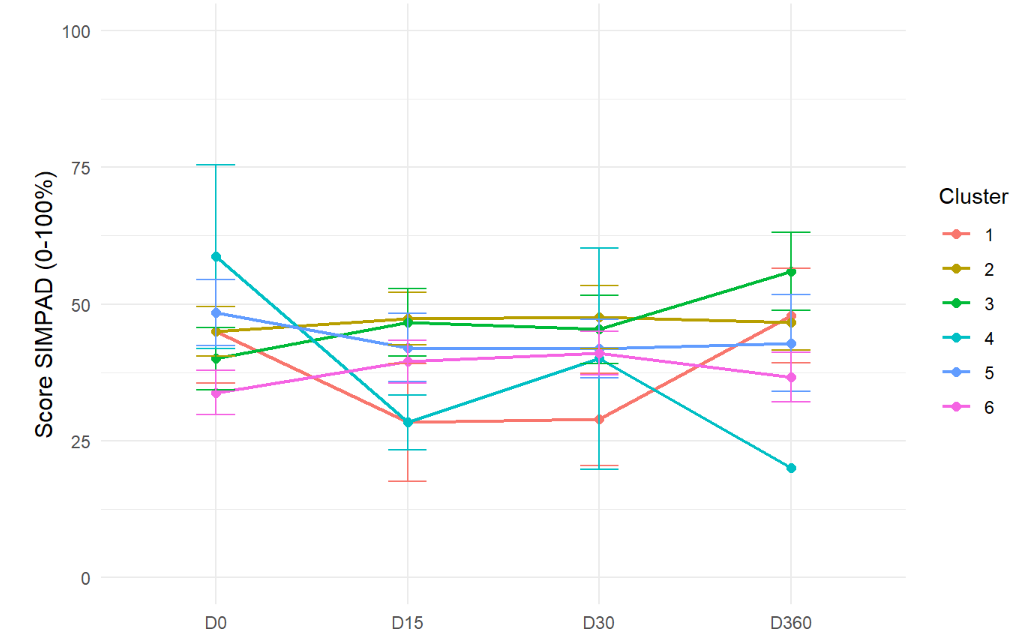  Dimension 4: Consideration at work |
| 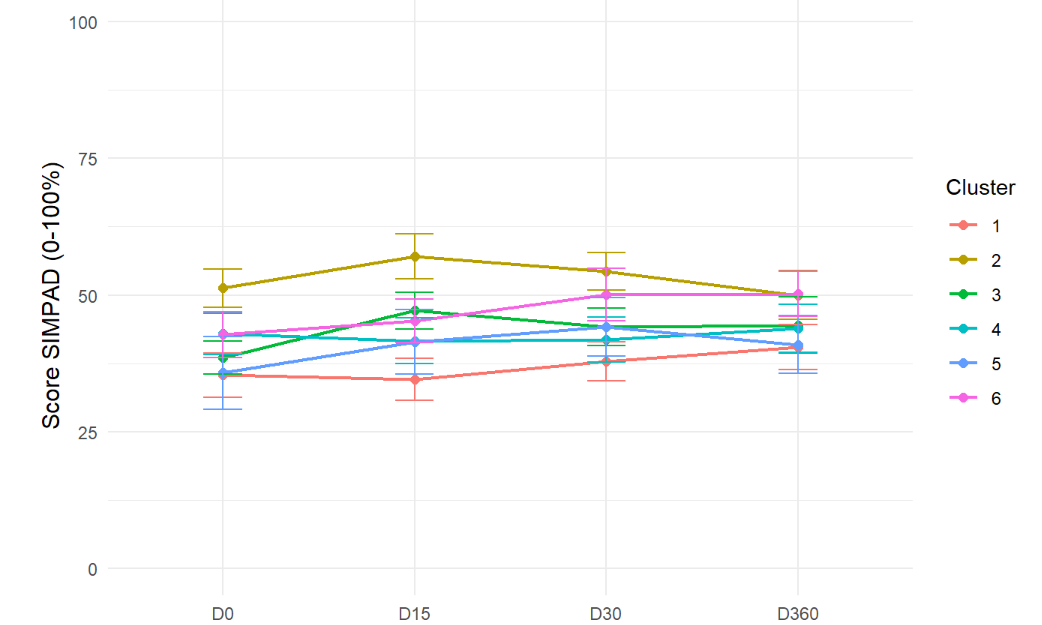  Dimension 5: The support of friends | 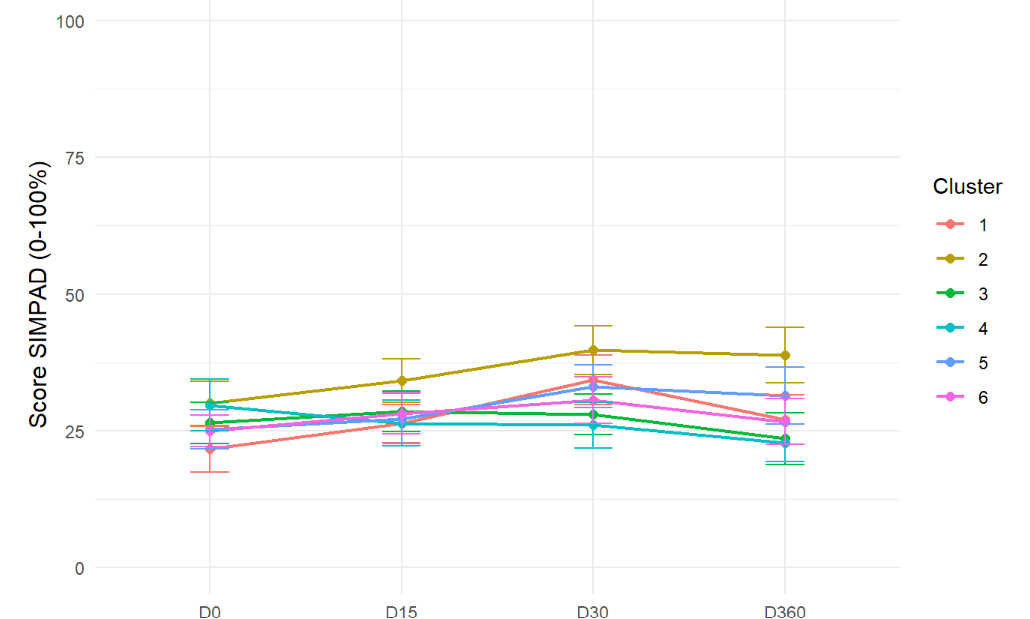  Dimension 6: The consideration of care team |
| 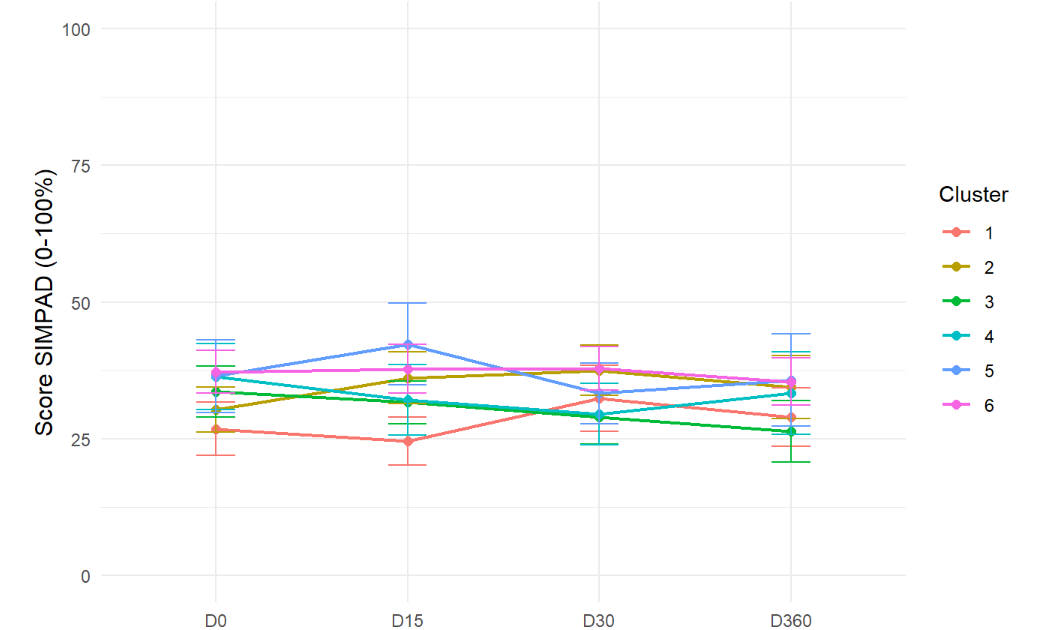  Dimension 7: Support from partner | 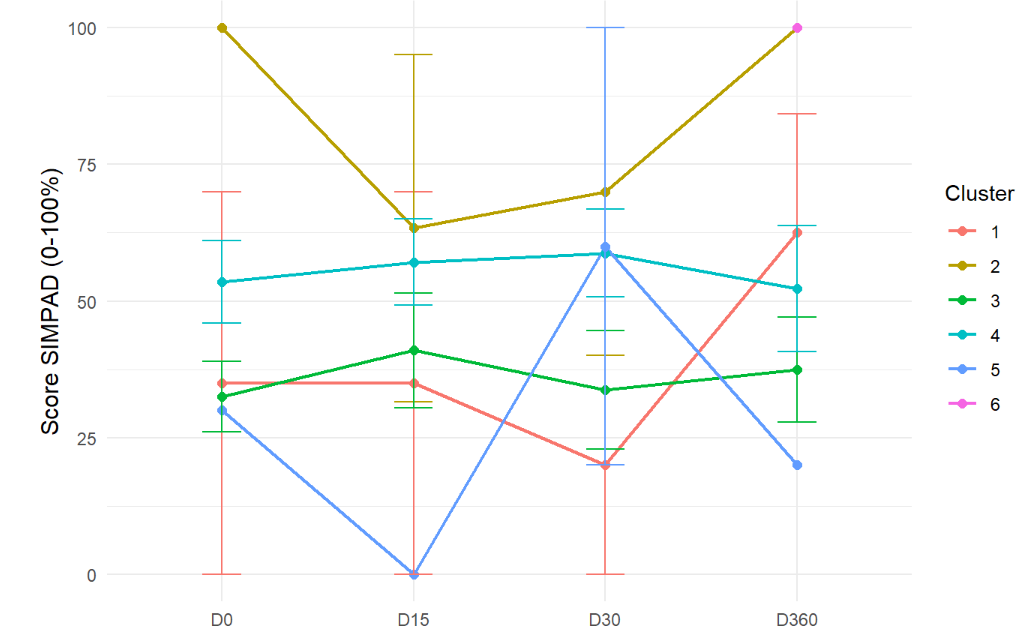  Dimension 8: Role as a grandparent |
| 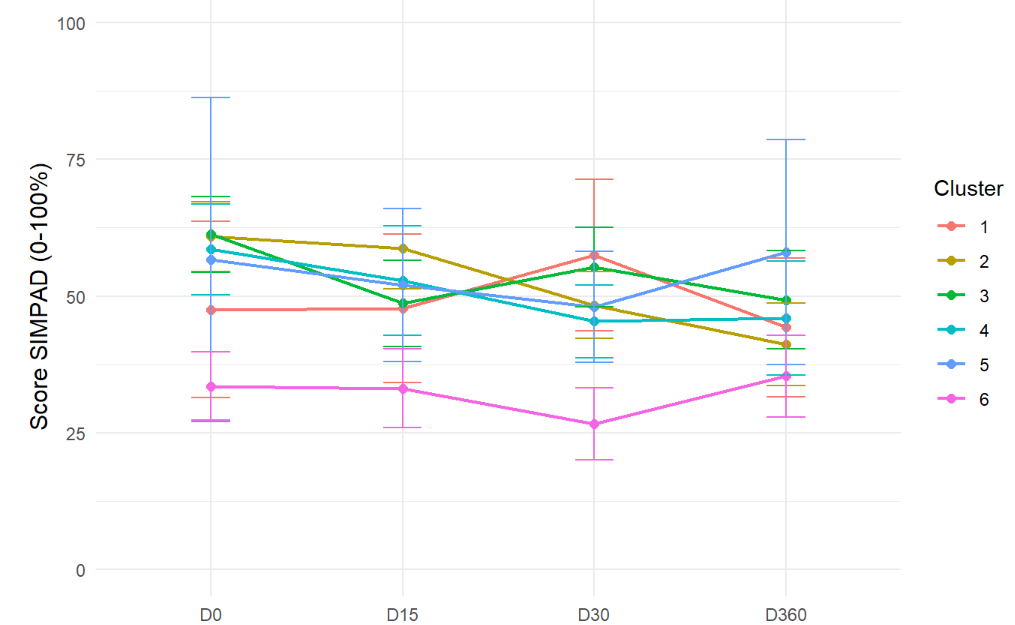  Dimension 9: Involvement in associative group | 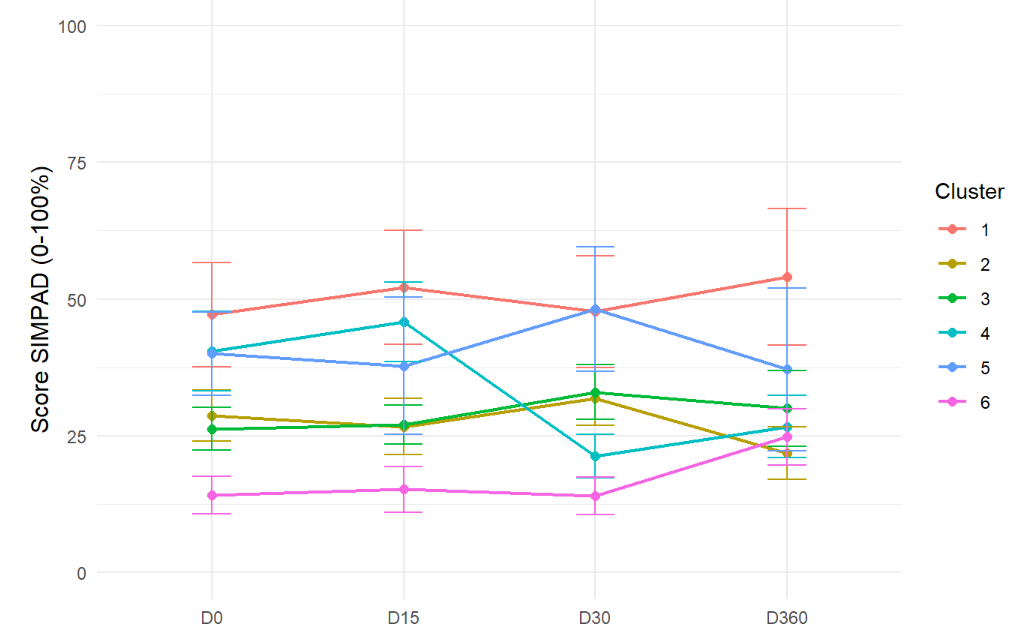  Dimension 10: : Involvement in activities (sports, leisure, travel) |

Footnote:Cluster 1 (middle-aged, on sick leave), Cluster 2 (younger, with occasional walking assistance), Cluster 3 (middle-aged, with personal activities), Cluster 4 (elderly, retired with leisure), Cluster 5 (younger, without activities), Cluster 6 (young, professionally active women).
