# Supplementary material for: A clustering-based approach to characterize autonomy profiles among multiple sclerosis patients: an application of the Qluster method in the FOCAL-MS2 study
Source: J Patient Rep Outcomes. 2026 May 25;10:125. doi: 10.1186/s41687-026-01074-5 (PMC13396066; doi:10.1186/s41687-026-01074-5)
Supplement: Supplementary file 2 — Supplementary Material 2 [file 41687_2026_1074_MOESM2_ESM.pdf]

Multiple Sclerosis Autonomy Scale

MSAS

Final version

Introduction to the questionnaire

The purpose of this questionnaire is to assess the **impact of multiple sclerosis on your daily life.** It is designed to give you the opportunity to describe how your personal or work activities and your social relationships have gone **over the past 4 weeks**.

It is important that you, and only you, answer the questions. This will allow your care team to better understand the impact of the condition on your life and offer you support to meet your needs.
Please complete it without getting help from a relative.

When completing this questionnaire, **think about the past 4 weeks** and select the answer that best corresponds to your current situation.

There are no right or wrong answers. We are interested in your answers, so **please answer in all honesty.**

Some questions may look similar, but they are all different. Some questions may not apply to you; however, please answer all the questions as best you can.

Over the past 4 weeks, has there been a significant event in your life that you think has had an impact on your multiple sclerosis?

- yes
- no

NOTE: if the “not applicable” option is checked, the associated impact questions on the topic will not be displayed in Section 2.

Section 1

Select how important the following topics are to you at the current time. If some questions do not apply to your <u>current</u> living situation, for example, if you do not have a job or if you are single or are not a grandparent, select “not applicable”.

***How important** is it for you…*	*Not at all important*									*The most important*
1. ...to be able to **participate in activities with your loved ones** (e.g., your children, your family, your friends, neighbors)?	0	1	*2*	*3*	*4*	*5*	*6*	*7*	*8*	*9*
6....to be able **to continue your work-related activities as you want to?**	0	1	2	3	4	5	6	7	8	9
this does not apply to me currently	☐									
13. ...to **be regarded by others at work**?	0	1	*2*	*3*	*4*	*5*	*6*	*7*	*8*	*9*
this does not apply to me currently	☐									
10. ...to **control the image you project to others**?	0	1	*2*	*3*	*4*	*5*	*6*	*7*	*8*	*9*
17. ...to be **able to get support from your friends**?	0	1	*2*	*3*	*4*	*5*	*6*	*7*	*8*	*9*
21. ...to **feel considered by your healthcare providers**?	0	1	*2*	*3*	*4*	*5*	*6*	*7*	*8*	*9*
24. ...to **feel supported by your partner**?	0	1	*2*	*3*	*4*	*5*	*6*	*7*	*8*	*9*
this does not apply to me currently	☐									
27. ...to **fulfill your role as a grandparent**?	0	1	*2*	*3*	*4*	*5*	*6*	*7*	*8*	*9*
this does not apply to me currently	☐									
30. ...to be **able to be involved in a club or an association in the way you want to**?	0	1	*2*	*3*	*4*	*5*	*6*	*7*	*8*	*9*

33. ...to be **able to take part in activities for yourself (sport, leisure activities, travel, etc.) in the way you want to**?	0	1	*2*	*3*	*4*	*5*	*6*	*7*	*8*	*9*

Section 2

1. Taking part in activities with others (4 items)

For the following items, think about the past 4 weeks (about 1 month) and especially the impact of MS on your life.

	not at all	*Very slightly*	*Slightly*	*moderately*	*significantly*	*totally*
2. *Over the past 4 weeks,* ***to what extent*** *have you* **felt you were living the life that you wanted**?						

To what extent do you agree or disagree with the following statements?	*Strongly disagree*	*disagree*	*Somewhat disagree*	*Somewhat agree*	*agree*	*Strongly agree*
3. *Over the past 4 weeks*, you have been **able to help your loved ones in the way you wanted to.**						
4. *Over the past 4 weeks*, **you have been able to move around in public places in the way you wanted to**.						
5. *Over the past 4 weeks,* you have been able to **maintain satisfactory relationships with your loved ones**.						

2. Your social and professional activities (3 items)

For the following items, think about the past 4 weeks (about 1 month) and especially the impact of MS on your life.

	never	*rarely*	*sometimes*	*often*	*very often*	*all the time*
7. *Over the past 4 weeks,* ***have there been times when*** you have **felt undervalued at work**?						
8. *Over the past 4 weeks,* ***have there been times when*** you were **worried about losing your job**?						
9. *Over the past 4 weeks,* ***have there been times when*** you have **felt you had to surpass your limits to do your job**?						

3. Controlling the image you project to others (4 items)

For the following items, think about the past 4 weeks (about 1 month) and especially the impact of MS on your life.

	never	*rarely*	*sometimes*	*often*	*Very often*	*all the time*
11. *Over the past 4 weeks,* ***have there been times when*** **you have restricted your activities in public places for fear of being judged because of your condition**?						
12. *Over the past 4 weeks,* ***have there been times when*** **you tried to hide your condition when you were in public places** (in the street, in a shop, etc.)?						

4. Feeling seen at work (4 items)

For the following items, think about the past 4 weeks (about 1 month) and especially the impact of MS on your life.

	not at all	*Very slightly*	*slightly*	*moderately*	*very much*	*totally*
14. *Over the past 4 weeks,* ***to what extent*** have **you felt supported by your employer**?						

	never	*rarely*	*sometimes*	*often*	*very often*	*all the time*
15. *Over the past 4 weeks,* ***have there been times when*** you **felt that your employer was going easy on you** (e.g., by protecting you or adapting your job)?						

To what extent do you agree or disagree with the following statements?	*Strongly disagree*	*disagree*	*Somewhat disagree*	*Somewhat agree*	*agree*	*Strongly agree*
16. *Over the past 4 weeks,* you have been able to **maintain satisfactory relationships with your work colleagues.**						

5. Support from your friends (3 items)

For the following items, think about the past 4 weeks (about 1 month) and especially the impact of MS on your life.

	never	*rarely*	*sometimes*	*often*	*very often*	*all the time*
18. *Over the past 4 weeks, have there been times when* you **felt supported by your friends**?						
19. *Over the past 4 weeks, have there been times when* you **felt that your friends were making allowances for you** (e.g., looking out for you, etc.)?						
20. *Over the past 4 weeks, have there been times when* you **felt your friends were affected by your condition**?						

6. How your opinion is taken into account by your healthcare team (2 items)

For the following items, think about the past 4 weeks (about 1 month) and especially the impact of MS on your life.

	not at all	*Very slightly*	*slightly*	*moderately*	*very much*	*totally*
21. *Over the past 4 weeks,* ***to what extent*** have you felt you were **taking an active part in decisions about your health**?						

	never	*rarely*	*sometimes*	*often*	*very often*	*all the time*
23. Over the past 4 weeks, have there been times when you **felt understood and supported by your healthcare providers**?						

7. Support from your partner (2 items)

For the following items, think about the past 4 weeks (about 1 month) and especially the impact of MS on your life.

To what extent do you agree or disagree with the following statements?	*Strongly disagree*	*disagree*	*Somewhat disagree*	*Somewhat agree*	*agree*	*Strongly agree*
25. *Over the past 4 weeks,* have you felt understood and supported*)* **by your partner?**						

	never	*rarely*	*sometimes*	*often*	*very often*	*all the time*
26. *Over the past 4 weeks,* ***have there been times when*** you felt your **partner was worried about your health**?						

8. Your role as a grandparent (2 items)

For the following items, think about the past 4 weeks (about 1 month) and especially the impact of MS on your life.

	not at all	*Very slightly*	*slightly*	*moderately*	*very much*	*totally*
28. *Over the past 4 weeks,* ***to what extent*** have you been able to **look after your grandchildren in the way you wanted to**?						
29. *Over the past 4 weeks,* ***to what extent*** have you been able to **be there for your grandchildren in the way you wanted to**?						

9. Your involvement in a community group (3 items)

For the following items, think about the past 4 weeks (about 1 month) and especially the impact of MS on your life.

	never	*rarely*	*sometimes*	*often*	*Very often*	*all the time*		*I was not involved in a club or an association*
31. *Over the past 4 weeks,* ***have there been times when*** you **felt useful as part of a club or an association**?								

	not at all	*Very slightly*	*slightly*	*moderately*	*very much*	*totally*		*I was not involved in a club or an association*
32. *Over the past 4 weeks,* ***to what extent*** have you been able to **maintain relationships with other members of a club or an association**?								

10. Your involvement in activities for yourself (sports, leisure activities, travel) (3 items)

For the following items, think about the past 4 weeks (about 1 month) and especially the impact of MS on your life.

To what extent do you agree or disagree with the following statements?	*Strongly disagree*	*disagree*	*somewhat disagree*	*Somewhat agree*	*agree*	*strongly agree*		*I have not done any activities for myself*
34. *Over the past 4 weeks*, you have **started, resumed, or continued a sporting or leisure activity.**								
35. *Over the past 4 weeks*, **your sporting or leisure activities have helped you to feel better.**								
