# Supplementary material for: A clustering-based approach to characterize autonomy profiles among multiple sclerosis patients: an application of the Qluster method in the FOCAL-MS2 study
Source: J Patient Rep Outcomes. 2026 May 25;10:125. doi: 10.1186/s41687-026-01074-5 (PMC13396066; doi:10.1186/s41687-026-01074-5)
Supplement: Supplementary file 3 — Supplementary Material 3 [file 41687_2026_1074_MOESM3_ESM.pdf]

Supplementary material number 9: Additional Patient questionnaire

1. Patient Characteristics

- **Living Situation:** Do you live alone? (Yes/No)
- **Home Assistance:** Is there someone (caregiver or professional) who regularly helps you at home with daily tasks like hygiene, transport, shopping, or meals (excluding housework)? (Yes/No)
- **Professional Situation:** Current status (Active, Unemployed, Medical leave/Disability, Student, Retired, Other).
- **Socioprofessional Category:** (Farmers, Business owners, Managers, Employees, Workers, Stay-at-home parent).
- **Education:** Highest degree obtained.
- **Family:** Number of dependent children and their ages; number of grandchildren.
- **Location:** Region of residence , housing type (apartment/house) , and size of town.
- **Access to Services:** Distance and mode of transport to the supermarket, pharmacy, GP, hospital, and work/school.
- **Leisure:** Participation in collective or personal leisure activities.

2. Autonomy and Satisfaction

- **Autonomy Anchor:** Overall, how capable do you feel of accomplishing activities that are most important to you (work, parenting, helping others)? (Scale: Never to Always).
- **Healthcare Professional (HCP) Interaction:** Since the last questionnaire, have you seen an HCP and discussed the results? Are you satisfied with that discussion?
- **Tool Experience:** Satisfaction with the tool (0-100 scale), visual presentation of results, and the time required to fill it out.
